# Environmental and Protection Effects of Shark‐Companion Associations Across Three Ocean Basins

**DOI:** 10.1002/ece3.73823

**Published:** 2026-06-09

**Authors:** Jett K. Walker, Jessica J. Meeuwig, Christopher D. H. Thompson

**Affiliations:** ^1^ Marine Futures Laboratory, School of Biological Sciences University of Western Australia Crawley Western Australia Australia; ^2^ Pristine Seas National Geographic Society Washington DC USA

**Keywords:** BRUVS, co‐decline, host, interspecies interactions, marine protection, pelagic ecology

## Abstract

Ecological associations such as mutualism, commensalism and parasitism strongly influence animal fitness and ecosystem structure, yet they remain poorly understood in the open ocean. Ecological interdependence means that the loss of one species can trigger co‐declines and accelerate biodiversity loss. Large, mobile animals often host smaller ‘companion’ species that gain benefits such as protection from predation, foraging opportunities, or host‐mediated transport through close association. We quantified companion‐host associations among seven shark species present across three ocean basins. This is the first large‐scale assessment to investigate how environmental conditions and marine protected‐area (MPA) status shape these associations. Data on species identity, abundance and length were obtained from a curated database derived from midwater Baited Remote Underwater Video Systems (BRUVS). Almost half of individual sharks hosted companions, with marked variation among species. Companions were most common on Australian blacktip sharks ( *Carcharhinus tilstoni* ) and least common on scalloped hammerheads ( *Sphyrna lewini* ). Companion presence was best predicted by sea surface temperature, salinity, wind speed and distance to shore, while companion abundance was predicted by primary productivity, wind speed and salinity. Several of these environmental factors are highly sensitive to climate change, potentially disrupting companion‐host associations. Partially protected areas (IUCN IV–VI) had a higher probability of companion occurrence than areas outside partial protection, whereas highly protected areas (IUCN I–II) supported greater companion abundance when companions were present. Declining host populations and changing environmental conditions may disrupt companion associations, removing the interspecies linkages and increasing the risk of co‐decline of dependent species. Effective protection of these associations will support the persistence of biodiversity and ecological processes in the open ocean.

## Graphical abstract

Companion species frequently associate with large marine hosts, yet the ecological drivers of these relationships remain poorly understood. Using a global dataset of midwater BRUVS deployments, we modelled the presence and abundance of shark‐companion associations across seven shark species and three ocean basins. Companion occurrence and abundance varied among host species and were predicted by environmental conditions and protection status, suggesting that changing ocean environments and declining shark populations may disrupt these interspecific associations.

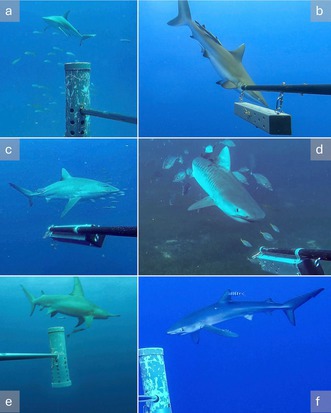

## 1 Introduction

Behavioural interactions and associations among animals are fundamental processes that influence population dynamics and ecosystem structure (Rahman and Candolin 2022; Sih et al. 2004; Schmitz et al. 2004). Interactions occur when species affect one another's fitness (Bronstein 1994; Sih et al. 2004) whereas associations describe repeated non‐random co‐occurrences within or among species that may or may not involve direct energetic or behavioural exchanges (Brunnschweiler et al. 2020; Gotelli and Graves 1996). These relationships range from exploitative interactions such as parasitism to facilitative associations including commensalism and mutualism (Bronstein 1994; Dallas and Cornelius 2015; Gayford 2024). Outcomes of interspecies behaviours rarely fit into rigid categories; instead forming a continuum of costs and benefits shaped by evolutionary history and environmental conditions (Bronstein 1994; Chamberlain et al. 2014; Rahman and Candolin 2022). Behavioural associations can alter ecological processes in ways that cascade through populations and influence ecosystem stability (Folke et al. 2004; Schmitz et al. 2004). For instance, cleaning symbioses among coral reef fishes reduce parasite loads and can influence the condition, abundance and survival of client species (Waldie et al. 2011; Grutter 1995). In contrast, competition among predators can suppress population growth (Segre et al. 2016) whereas kleptoparasitic behaviour can divert foraging effort (Shealer et al. 2004).

Sharks serve as focal hosts for a range of smaller companion species in marine ecosystems (Norman et al. 2021; Gayford 2024). Hosts are defined here as mobile marine animals that provide living surfaces, shelter, or transport for other species, consistent with broader definitions of symbiotic associations (Bronstein 1994; Gayford 2024). Companions are smaller individuals that associate closely with hosts and may gain benefits such as protection from predators, enhanced foraging opportunities, or transport, as observed in a range of marine host–associate systems (Norman et al. 2021; Gayford 2024). Companion‐host associations are well documented across diverse marine systems, including cleaner–client interactions (Caves 2021), turtle and marine mammal symbioses (e.g., remoras, barnacles, whale lice) (Carrillo et al. 2015; Lehnert et al. 2021; Sazima and Grossman 2006), and fish–invertebrate commensalisms (e.g., goby–shrimp) (Karplus and Thompson 2011). Associations between sharks and remoras ( *Remora remora* ) and pilotfish ( *Naucrates ductor* ) are two well‐known examples (Brunnschweiler et al. 2020; Fuller and Parsons 2019). Remoras attach with an adhesive disc (O'Toole 2002), using the host for transport and feed on ectoparasites or food scraps (Norman et al. 2021). Free‐swimming companions such as pilotfish, jacks and trevallies benefit from hosts via access to food and reduced predation risk (Fontes et al. 2023; Fuller and Parsons 2019). Benefits in companion‐host associations are often asymmetrical, with little evidence that sharks gain consistent benefits by hosting companions (Brunnschweiler et al. 2020; Fontes et al. 2023). These associations are therefore most often described as commensal but may shift towards parasitism depending on ecological context (Brunnschweiler et al. 2020; Gayford 2024). Descriptions of companion‐host associations in pelagic environments are often based on studies of charismatic megafauna such as cetaceans (Flammang et al. 2020), turtles (Sazima and Grossman 2006) and sharks (Gayford 2024).

Environmental variables likely influence companion‐host associations, but the extent to which they predict these associations remains poorly understood. Environmental conditions are known to influence species interactions across ecological systems by altering encounter rates, spatial overlap and interaction strength (Tylianakis and Morris 2017). Salinity and temperature gradients can structure habitat boundaries and influence species co‐occurrence. In marine systems, these gradients often manifest as ocean fronts, where convergent flow concentrates organisms (Wolanski and Hamner 1988; Woodson and Litvin 2015). for example, fronts defined by temperature and salinity gradients have been shown to aggregate predators and prey, resulting in substantial spatial overlap and increased potential encounter rates (Woodson and Litvin 2015). Therefore, we expect companion‐host associations to increase in areas with stronger temperature and salinity gradients. Distance to shore and primary productivity influence the structure of large pelagic wildlife distributions globally (Block et al. 2011; Letessier et al. 2019). These same factors are also likely to influence encounter rates between hosts and companions by structuring species distributions and aggregations (Block et al. 2011; Baudena et al. 2021; Chamberlain et al. 2014). Large pelagic predators have been shown to aggregate in regions of elevated productivity where prey availability is enhanced (Block et al. 2011) while lower fish abundances in offshore waters may reduce companion‐host encounters (Yan et al. 2025; Baudena et al. 2021). Wind‐driven processes further contribute to these dynamics by generating mixing and upwelling that enhance local productivity and concentrate prey resources (Barlow et al. 2021; Croll et al. 2005) for example, wind‐driven upwelling has been shown to bring nutrient‐rich waters to the surface, increasing primary production and subsequently supporting higher densities of prey and marine predators (Barlow et al. 2021). Increasing intensity of such processes are expected to increase the probability of interactions between hosts and companions by enhancing co‐occurrence and encounter frequency. Many of these environmental variables are sensitive to climate change (Garcia‐Soto et al. 2021) meaning ecological associations that depend on them may be vulnerable to shifting ocean conditions (Colwell et al. 2012; Strona and Bradshaw 2018).

Host traits may shape companion–host associations. Larger sharks are expected to carry more companions because they offer greater surface area for attachment, which scales predictably with body size (Gayford et al. 2025), although empirical support for size effects on companion abundance remains mixed (Gayford 2024; Moir et al. 2010). Differences among shark species could further contribute to variation in associations, as species differ markedly in morphology and behaviour, which may influence how frequently they encounter or retain companions. For example, differences in diving behaviour (Carbonara et al. 2024) and migratory movements may affect encounter rates between hosts and companions (Nathan et al. 2008). Patterns of association may also reflect the degree of host specialisation. Companions that associate with multiple host species may be less sensitive to host declines, whereas those dependent on a single host may face an elevated risk of co‐decline (Colwell et al. 2012).

Populations of oceanic sharks have fallen by more than 70% over the past half‐century (Pacoureau et al. 2021), largely due to overfishing and resource extraction, with climate change acting as an additional stressor that may compound these declines (Hatton et al. 2025; Krishna et al. 2025). Despite these declines, the ecological consequences of host loss for companions remain largely unexplored (Fuller and Parsons 2019; Gayford 2024). The depletion of host populations may remove ecological niches on which companions rely. Reduced host availability may increase predation risk for companions that use sharks as mobile shelter or scavenging opportunities (Fontes et al. 2023; Gayford 2024; Sazima and Grossman 2006). Marine Protected Areas (MPAs) are central to mitigating biodiversity loss and rebuilding populations of large marine species (Lester et al. 2009; Sala and Giakoumi 2017). Highly protected areas (IUCN I–II), defined as no take reserves (Day et al. 2012), prohibit extraction and deliver stronger ecological outcomes than partially protected areas (IUCN IV–VI), where ongoing exploitation often limits population recovery (Costello and Ballantine 2015; Turnbull et al. 2021). Although many MPAs remain residual and are placed in regions of low fishing pressure or low baseline fish densities, effective protection can restore trophic structure and promote the recovery of ecological interactions (Devillers et al. 2014; Horta e Costa et al. 2025). However, many pelagic shark species undertake large‐scale movements and frequently cross MPA boundaries (Jacoby et al. 2020), which may reduce differences in companion‐host associations between protected and unprotected areas. At the same time, MPAs can still enhance these associations indirectly by increasing the abundance and size structure of host populations (Lester et al. 2009; Horta e Costa et al. 2025), thereby supporting companion species that rely on hosts for refuge, food, or transport (Norman et al. 2021; Gayford 2024).

A curated global dataset of Baited Remote Underwater Video Systems (BRUVS) was used to quantify companion–host associations. BRUVS provide a non‐destructive, standardised approach for surveying marine fauna across broad spatial scales (Bouchet et al. 2018; Buschmann et al. 2026; Harvey et al. 2021) and are well suited to detecting associations among mobile species. Midwater BRUVS extend this approach into pelagic environments, offering consistent and replicable observations of free‐swimming species that are otherwise challenging to sample (Letessier et al. 2013). The dataset spans deployments across the Indian, Pacific and Atlantic Oceans and supports an assessment of ecological and conservation factors influencing companion–host associations. Here, sharks were selected as focal hosts because they allow these interactions to be examined in large, mobile species that span broad environmental gradients while also being well represented in the BRUVS dataset. The selected species span multiple families and ecological traits, allowing evaluation of interspecific variation in companion‐host associations. In this study, we compare companion presence/absence and abundance among host species, identify environmental and host‐level predictors of these associations, and test whether protection status influences companion presence/absence and abundance. Environmental variables were selected based on their known roles in structuring species distributions, encounter rates, and aggregation processes. We hypothesise that (a) companions show non‐random associations with host species, (b) environmental variables can support a predictive framework for host‐companion associations through their effects on encounter rates and species overlap, and (c) highly protected areas support greater frequency and abundance of companions relative to partially protected or unprotected areas. Empirical data on companion ecology and behaviour remain sparse, despite growing evidence from other interspecies systems that the loss of hosts can trigger secondary co‐decline among dependant species (Strona and Bradshaw 2018). Companion‐host associations must be understood to anticipate how ongoing declines in marine megafauna may cascade through pelagic ecosystems.

## 2 Materials and Methods

### 2.1 BRUVS Dataset and Video Analysis

Observations of sharks and companions were obtained from a global database curated by the Marine Futures Lab (https://www.meeuwig.org/). Identifications, abundances, and sizes compiled in this database were obtained using midwater BRUVS deployed following standardised protocols (Bouchet et al. 2018). Each BRUVS consisted of a rigid frame with two high‐definition video cameras (GoPro, recording 1080p 30 frames per second) in waterproof housings mounted on a central base bar 80 cm apart and angled inwards by 8°, a perpendicular 1.8 m long bait arm, and an upright bar connected to a weight and surface line. A ~45 cm long perforated PVC canister containing ~1 kg of oily pelagic fish was attached at a fixed distance on the bait arm perpendicular to the centre of the two cameras. Stereo cameras were calibrated prior to deployment to enable accurate length measurements from paired camera footage (Harvey and Shortis 1998). BRUVS were deployed in a longline configuration in sets of three to five rigs, suspended from a surface buoy at a depth of 10 m, and each separated by 200 m of horizontal line. Units were left unattended for a minimum soak time of 120 min during daylight hours to minimise the influence of crepuscular behavioural changes of fish.

**FIGURE 1:**
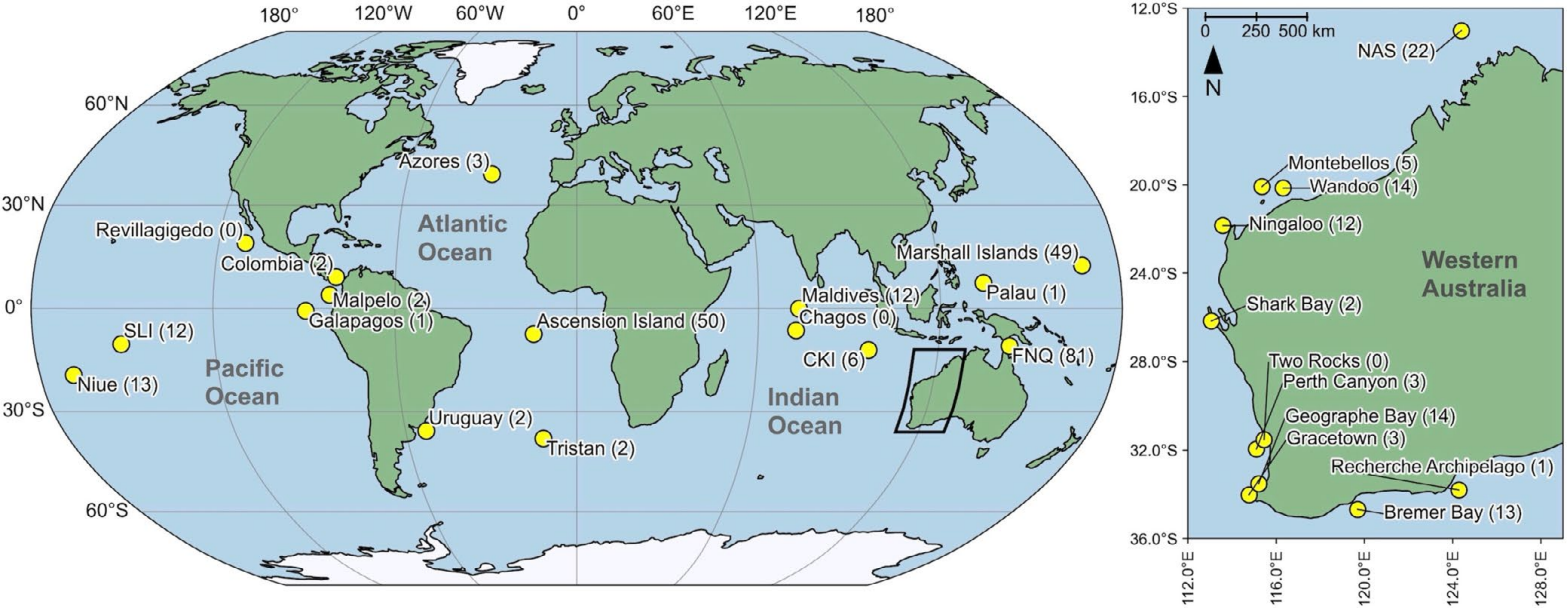
Map showing the global distribution of 27 sampling locations used in this study. Yellow circles indicate localities, with the number of deployments with companion associations provided in parentheses. The main panel depicts sites distributed across the Pacific, Atlantic and Indian Oceans, while the inset shows the spatial arrangement of localities along the coast of Western Australia. Several sites are abbreviated for clarity: FNQ, Far North Queensland; CKI, Cocos (Keeling) Islands; SLI, Southern Line Islands; NAS, Northwest Australian Shelf.

At the time of analysis, the midwater BRUVS dataset contained 188,984 individual animals from 113 families, recorded across 8827 midwater deployments across 48 global locations between 2014 and 2024 (Figure 1; Table S1). A list of candidate companion and host species was first generated through a literature review to identify reported associations. The database was filtered to retain deployments containing records of both companions and hosts in the same video. Candidate hosts were then selected using two criteria: (1) the candidate host was present on at least 40 deployments; and (2) one or more candidate companions were previously recorded in the same deployment. To ensure sufficient replication and comparability across taxa, we restricted our final selection of hosts to seven shark species that were well represented in the database spanning three ecologically distinct families, Carcharhinidae, Galeocerdonidae and Sphyrnidae (da Silva et al. 2021; Gallagher and Klimley 2018; Speed et al. 2010). Data from 2767 BRUVS deployments were filtered to include all records, regardless of companion presence for: Australian blacktip sharks ( *Carcharhinus tilstoni* ), blue sharks ( *Prionace glauca* ), copper sharks ( *Carcharhinus brachyurus* ), grey reef sharks ( *Carcharhinus amblyrhynchos* ), tiger sharks ( *Galeocerdo cuvier* ), great hammerheads ( *Sphyrna mokarran* ), and scalloped hammerheads ( *Sphyrna lewini* ). A subset of 373 deployments was selected by stratified random sampling to balance host representation across locations (Iqbal et al. 2024; Table S1).

**FIGURE 2:**
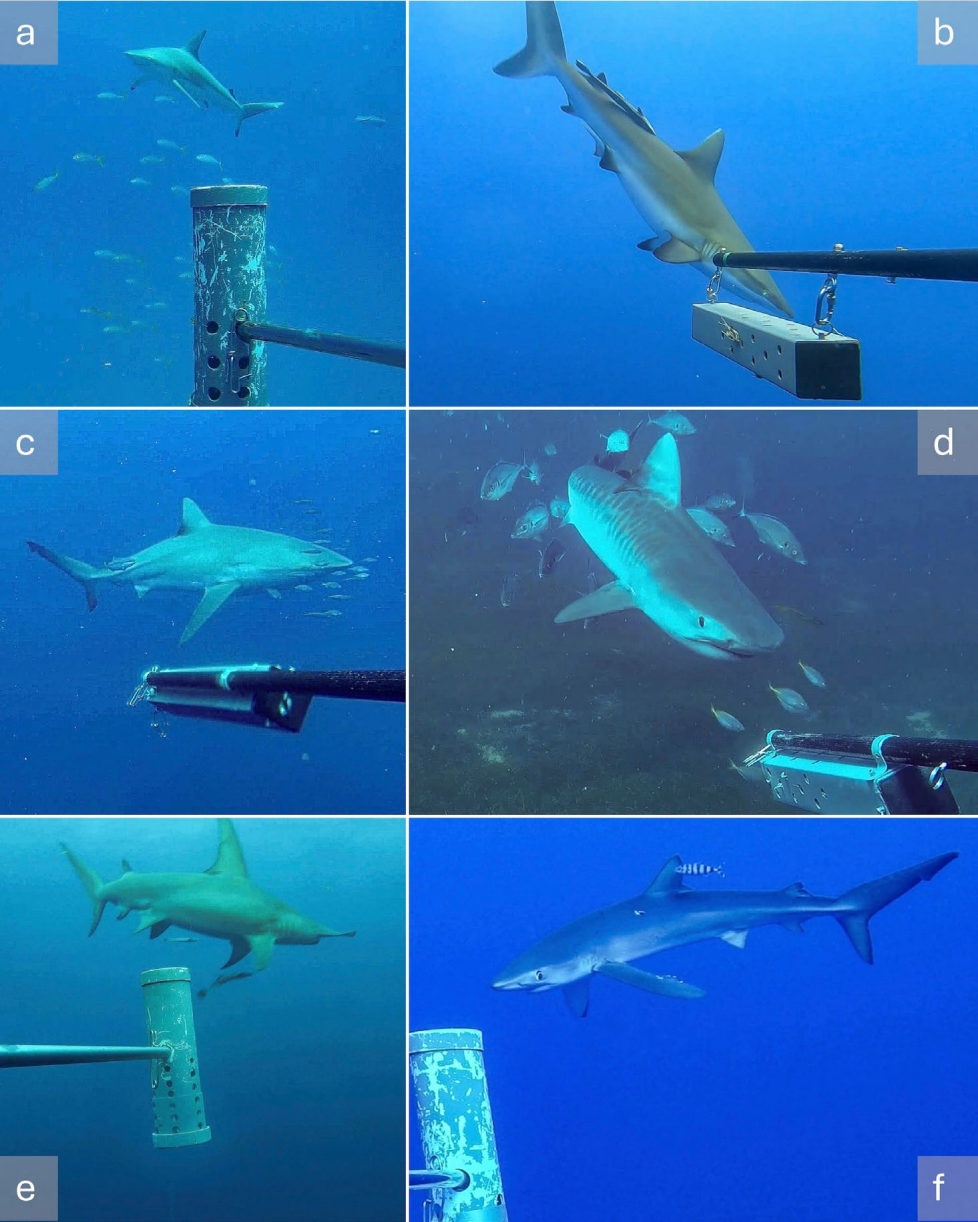
Example images of host–companion associations recorded on BRUVS. Panels illustrate different shark species observed with companions. (a) Australian blacktip shark ( *Carcharhinus tilstoni* ) with yellowtail scad ( *Atule mate* ); (b) grey reef shark ( *Carcharhinus amblyrhynchos* ) with a remora ( *Remora remora* ); (c) Copper shark ( *Carcharhinus brachyurus* ) with unidentified scads (*Decapterus* sp.) and a rainbow runner ( *Elagatis bipinnulata* ); (d) tiger shark ( *Galeocerdo cuvier* ) with a large group of diverse companions; (e) great hammerhead ( *Sphyrna mokarran* ) accompanied by remora; (f) Blue shark ( *Prionace glauca* ) with pilotfish ( *Naucrates ductor* ).

Companion attributes were linked to the corresponding host record to generate a combined dataset of host–companion associations. Companions were classified as suction or free‐swimming. Suction companions, such as remoras, attach to hosts using an adhesive disc (Brunnschweiler et al. 2020). Free‐swimming companions, such as pilotfish, maintain proximity without contact (Fontes et al. 2023). Each shark received a unique identifier to distinguish individuals when multiple hosts appeared within a video. Video annotation and fork length measurements were conducted using EventMeasure (www.seagis.com.au). Data were extracted from BRUVS videos in which a host was clearly visible (Figure 2). Observed companions were recorded swimming within five metres of a host for the entire duration of an observation lasting at least 10 s and were required to enter and exit the camera field of view with the host, indicating consistent spatial association rather than incidental proximity. For each host, we recorded species identity, fork length (cm), number of companions and companion species richness.

Species‐specific associations were documented using a bipartite network constructed from the combined dataset of individual companion‐host records. Each band represented the strength of an association between a host and a companion species. Band width was weighted by the frequency of co‐occurrence across all deployments. Companion nodes were colour‐coded by attachment mode (suction or free‐swimming) to distinguish functional groups. The network was produced in R (version 4.5.0) using the ggalluvial and ggplot2 packages (Brunson 2020; Wickham 2016). This approach provided a graphical representation of the overall structure of associations and the degree of host‐companion specificity.

The probability that a companion occurred with a host was modelled using a binomial logistic regression (Hosmer Jr. et al. 2013; Quinn and Keough 2002). The model estimated the probability of observing companions for each host species, allowing pairwise comparisons of companion occurrence among host species. Odds ratios were calculated as the ratio of the odds of companion presence between two host species (e.g., the odds of companions on tiger sharks relative to those on blue sharks), where values greater than one indicated a higher relative likelihood of hosting companions. Pairwise differences were tested for significance using Wald tests, and 95% confidence intervals and *p*‐values quantified statistical uncertainty (Menard 2002).

### 2.2 Modelling Companion‐Host Associations

Companion presence and abundance were modelled using a negative binomial hurdle model. The hurdle model addressed two key features of our dataset: a high proportion of zeros (53% of records) and overdispersed positive counts. A negative binomial hurdle formulation was used because zeros in the model represent true absence of companions rather than a separate latent process generating structural zeros. Zero‐inflated models assume that some observations belong to a latent state in which non‐zero counts cannot occur. Because all sharks in our dataset could potentially host‐companions, this assumption was not biologically justified. The hurdle approach used here is described as a two‐part process suited to ecological count data by Potts and Elith (2006) and Welsh et al. (1996). The first component estimated occurrence probability. The second component was a zero‐truncated count model that estimated the number of companions given presence (*n* > 0). These two parts treat the processes generating presence/absence and variation in abundance conditional on presence as potentially separate processes (Potts and Elith 2006). Prior to model fitting, two sharks with exceptionally high companion counts (70 and 68 individuals) were excluded. These values represent rare but biologically plausible aggregation events. However, they exerted disproportionate influence on model dispersion and reduced predictive performance during cross validation. These two outliers were removed as the objective of the model was to characterise general patterns of host‐companion associations rather than rare aggregation events.

Predictors of companion abundance were selected from variables commonly used to model large pelagic species distributions (Letessier et al. 2019). A suite of continuous variables was derived from a database curated by the MARBEC laboratory at the University of Montpellier France (https://umr‐marbec.fr/en/) (Table 1). Environmental variables were matched to BRUVS deployments using monthly‐averaged conditions preceding each observation to better align predictor scale with the ecological processes influencing companion‐shark occurrence. Instantaneous environmental measurements associated with BRUVS deployments represent short‐term conditions that are often highly variable and may obscure underlying habitat relationships, particularly for mobile predators responding to temporally integrated thermal and productivity fields. The use of temporally averaged environmental covariates to reduce noise and improve ecological inference is standard in marine species distribution modelling and pelagic ecology (e.g., Robinson et al. 2011; Brodie et al. 2018). Potential predictor variables were: seabed depth (m), distance to shore (km), chlorophyll concentration (mg m^−3^), primary production (mg m^−2^), salinity (ppt), dissolved oxygen (mmol m^−3^), sea surface temperature (°C), zonal wind component (windU; m s^−1^), meridional wind component (windV; m s^−1^), and distance to the nearest port (km) (Table 1). Continuous predictors with large ranges (> 1 order of magnitude) were log_10_‐transformed to aid model convergence and facilitate comparison of effect sizes across predictors. Multicollinearity among continuous predictors was evaluated using variance inflation factors (VIFs). Only predictors with VIF values < 2 were retained to reduce redundancy and improve inference (Zuur et al. 2010). A conservative threshold was used because even moderate collinearity can inflate uncertainty and mask weak ecological effects (Zuur et al. 2010). Host fork length (cm) was also included to evaluate whether host size influenced companion presence and/or abundance. A Pearson correlation test was additionally used to evaluate the bivariate relationship between host fork length and companion abundance (Quinn and Keough 2002).

**TABLE 1:** Geomorphological, environmental and proxies of human impact, with their abbreviations (abbrev), units of measurements (units), the description of variable, and the data source as well as the time scale of data collection.

Variable type	Variable	Abbrev; Units	Min	Max	Mean	Description	Source
Geomorphological	Seabed depth	Depth; m	15	4054	508	Vertical distance from the sea surface to the seafloor, representing habitat depth.	GEBCO^a^
	Distance to shore	DTS; km	0.8	356.5	57.8	Minimum horizontal distance from each sampling point to the nearest coastline.	Copernicus
Environmental	Chlorophyll concentration	Chl; mg m^−3^	27.2	1103.5	221.9	Concentration of phytoplankton pigments in surface waters, used as a proxy for biomass of phytoplankton.	Copernicus; daily
	Primary production	PP, mg m^−2^ day^−1^	197.2	2490.2	699.2	Rate of carbon fixation by photosynthetic organisms, reflecting energy availability at the base of the food web.	Copernicus; monthly
	Salinity	Sal; ppt	31.8	36.6	34.7	Concentration of dissolved salts in seawater, typically expressed in parts per thousand.	Copernicus; daily
	Dissolved oxygen	Oxy; mg L^−1^	195.8	254	209.2	Amount of oxygen dissolved in the water column, influencing aerobic metabolism and habitat suitability.	Copernicus; daily
	Sea surface temperature	SST; C	18.2	29.8	26.8	Temperature of the ocean's uppermost layer, a key driver of species distributions and metabolic rates.	Copernicus; monthly
	Zonal wind component	windU; m s^−1^	−9.1	10.5	−2.8	East–west wind velocity component; positive values indicate eastward winds, negative values westward.	Copernicus; hourly
	Meridional wind component	windV; m s^−1^	−6.7	10.4	1.7	North–south wind velocity component; positive values indicate northward winds, negative values southward.	Copernicus; hourly
Human influence proxy	Distance to port	DTP; km	1.1	655.2	227.1	Minimum distance from each sampling point to the nearest port, used as a proxy for human influence and fishing pressure	Global Fishing Watch

^a^ General Bathymetric Chart of the Oceans.

Protection was classified by three categories: IUCN I‐II, IUCN IV‐VI and areas outside designated MPAs. Two dummy variables were generated to represent these categories. The first dummy variable (DVHP) coded samples as inside (1) or outside (0) IUCN I–II areas. The second dummy variable (DVPP) coded samples as inside (1) or outside (0) IUCN IV–VI areas (Dudley 2008, https://portals.iucn.org/library/node/9243). The protection category was determined by overlaying deployment coordinates with protected‐area shapefiles corresponding to the time of sampling.

The predictive model was developed using a multi‐stage approach to maximise efficiency and accuracy. A pre‐screening phase was used to reduce computational load using the hurdle() function in the pscl R package (Zeileis et al. 2008). The pre‐screening process evaluates predictor contributions to model fit (Zhou and Lin 2017, 1849). Each candidate model was tested on a random subsample of the dataset and the 600 best‐ranked models were retained. Preliminary model runs indicated that increasing the cap beyond 600 did not alter top‐ranked models but greatly increased computation time. Models were ranked by corrected Akaike's Information Criterion (AICc) following Burnham and Anderson (2002) and Lester et al. (2022). Retained models were refitted in parallel using the glmmTMB R package and re‐analysed with AICc (Brooks et al. 2017). The final model showed the lowest AICc, indicating the best fit to the data. Model performance was compared to the null model to assess overall goodness of fit. A 10‐fold cross‐validation (CV) on the final hurdle model was completed to evaluate predictive performance. The dataset was randomly partitioned into 10 equal folds using the caret R package (Kuhn 2008). The model was trained on 90% of the data and evaluated on the remaining 10%. Predictive performance was assessed using mean absolute error (MAE) for abundance predictions and area under the receiver operating characteristic curve (AUC) for presence–absence (Hyndman and Koehler 2006; Kuhn 2008). Calibration was evaluated using out‐of‐fold predictions grouped into 10 equal‐frequency groups and comparing mean predicted and observed abundances with 95% confidence intervals (Kuhn 2008). All analyses were performed in R (version 4.5.0; R Core Team).

Model‐based predictions were generated to evaluate how protection status and continuous predictors influenced companion presence and abundance. Predictions followed post‐estimation methods outlined by Lenth (2016) and Arel‐Bundock et al. (2024). The fitted hurdle model was used to estimate the occurrence probability and conditional mean abundance for each variable of interest. Model effects were visualised using partial‐effects plots for each focal predictor. Plots were generated by varying the focal predictor across its observed range while holding all other variables at their mean (Lester et al. 2022). Protection effects were estimated for unprotected, partially protected (IUCN IV–VI), and highly protected (IUCN I–II) categories. The ten‐fold cross‐validation predictions were pooled across folds and visualised with a calibration plot. For each bin, the mean predicted abundance was plotted against the mean observed abundance with 95% confidence intervals. Plots were generated in R using ggplot2, patchwork, and supporting packages (Pedersen 2025; Wickham 2016). Uncertainty in model predictions was quantified with 2000 draws from a multivariate normal distribution from the model's coefficient covariance matrix, producing 95% confidence intervals. Presence/absence coefficients were converted to odds ratios, and abundance coefficients were expressed as ratios of conditional means (Arel‐Bundock et al. 2024). Odds ratios quantify changes in the relative likelihood of companion presence/absence between protection levels, whereas ratios of conditional means describe multiplicative changes in expected companion abundance when present, as the count model estimates effects on expected counts rather than probabilities.

## 3 Results

### 3.1 Patterns of Companion‐Host Associations, Who Swims With Whom

A total of 699 individual hosts from seven shark species were recorded across the subset of 373 BRUVS deployments. These observations formed the dataset used to quantify companion–host associations and fit the hurdle models described in the statistical methods. The number of individual hosts per shark species ranged from 37 to 278 individuals (Table 2). Nearly half (46.5%) of all hosts had at least one companion. Mean host species fork length ranged from 101 cm (Australian blacktip sharks ( *Carcharhinus tilstoni* )) to 256 cm (tiger sharks ( *Galeocerdo cuvier* )). The smallest and largest individual hosts were a 47 cm blue shark ( *Prionace glauca* ) and a 5.6 m tiger shark (Table 2). Forty percent of hosts occurred inside protected areas, including highly protected areas (IUCN I–II) and partially protected areas (IUCN IV–VI), with the remainder observed outside MPAs. Grey reef (*Carcharhinus amblyrhinchos*), copper ( *Carcharhinus brachyurus* ), blue, and scalloped hammerhead sharks ( *Sphyrna lewini* ) were more abundant outside MPAs, whereas Australian blacktip, tiger and great hammerhead sharks ( *Sphyrna mokarran* ) were proportionally more common inside MPAs (Table 2).

**TABLE 2:** Hosts and companions observed on midwater BRUVS by family, scientific name, common name, with number of individuals (*n*‐ind), the number of deployments the species was observed in (*n*), minimum fork length (minFL; cm), maximum fork length (maxFL; cm), mean fork length (meanFL; cm), standard deviation of fork length for each species (SD; cm), the percent of individuals observed in protected areas (%Prot), and the percentage of hosts with companions (%Com).

Family	Species	Common name	*n*‐ind	*n*‐dep	minFL	maxFL	meanFL	SD	%Prot	%Occ
**Host**										
Carcharhinidae	*Carcharhinus amblyrhynchos*	Grey reef shark	278	97	60	337	117	34.7	28%	21%
	*Carcharhinus brachyurus*	Copper shark	50	40	79	277	174	53.1	34%	42%
	*Carcharhinus tilstoni*	Australian blacktip Shark	74	41	61	158	101	22.5	84%	72%
	*Prionace glauca*	Blue shark	72	69	47	344	174	54.5	11%	84%
Galeocerdonidae	*Galeocerdo cuvier*	Tiger shark	56	54	110	567	256	98.1	57%	83%
Sphyrnidae	*Sphyrna lewini*	Scalloped hammerhead	132	45	68	352	137	48.3	45%	6%
	*Sphyrna mokarran*	Great hammerhead	37	35	86	369	211	59.6	73%	49%
**Companion**										
Carangidae	*Alepes* sp.	Alepes scads	3	1	32	38	35	3.2	33%	—
	*Atule mate*	Yellowtail scad	382	29	5	29	20	6.4	98%	—
	*Caranx crysos*	Blue runner	1	1	—	—	—	—	0%	—
	*Decapterus macarellus*	Mackerel scad	33	11	5	36	16	6.6	85%	—
	*Decapterus* sp.	Scads	158	16	4	3	14	5.9	85%	—
	*Elagatis bipinnulata*	Rainbow runner	3	2	20	80	26	31.6	33%	—
	*Gnathodon speciosus*	Golden trevally	3	1	6	7	7	0.3	100%	—
	*Naucrates ductor*	Pilot fish	97	56	5	58	25	10.9	20%	—
	*Pseudocaranx dentex*	White trevally	10	1	9	12	13	5.1	100%	—
	*Pseudocaranx georgianus*	Silver trevally	26	2	8	17	11	2.4	100%	—
	*Pseudocaranx* sp.	Trevally	12	4	4	19	11	6.0	100%	—
	*Pseudocaranx wrighti*	Skipjack trevally	1	1	—	—	—	—	100%	—
	*Selar crumenophthalmus*	Bigeye Scad	1	1	—	—	—	—	0%	—
	*Seriola lalandi*	Yellowtail amberjack	1	1	—	—	—	—	100%	—
	*Seriola* sp.	Amberjacks	1	1	14	14	14	—	100%	—
	*Trachurus* sp.	Mackerel	3	1	12	16	14	2.1	100%	—
Echeneidae*	*Echeneis naucrates*	Live sharksucker	129	87	4	67	23	29.8	70%	—
	*Remora albescens*	White suckerfish	2	2	7	8	NA	0.8	0%	—
	*Remora remora*	Remora	133	64	2	55	14	7.9	7%	—

*Note:* Dashes ‘–’ indicate taxa for which length measurements were not available. * Indicates suction companions, all other companions are free‐swimming.

**FIGURE 3:**
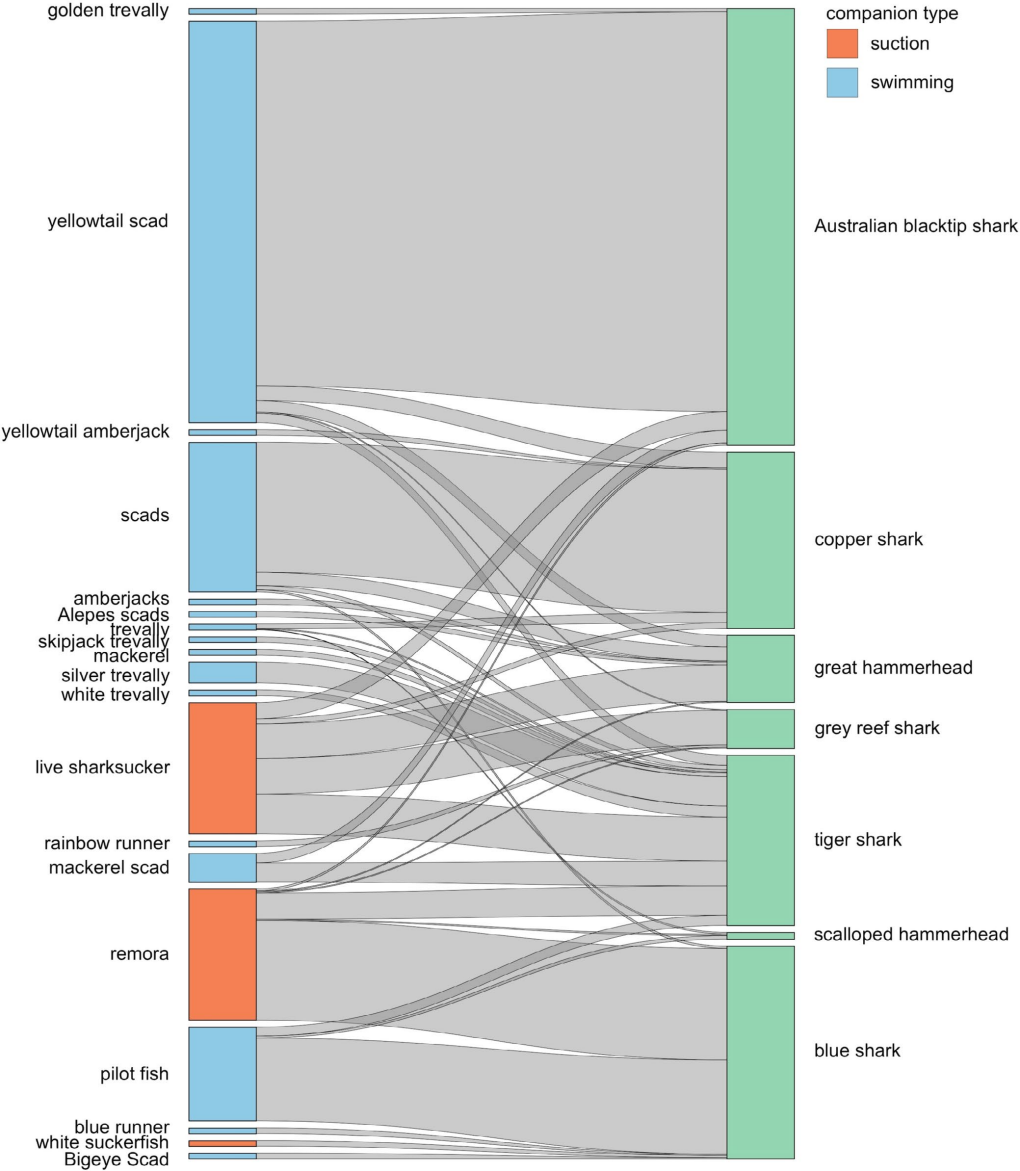
Bipartite network showing associations between host shark species (right) and companion species (left). Each line represents an observed association between a host and a companion with line thickness indicating the number of observations. Companion species are colour‐coded by attachment mode: Suction‐based (orange) or free‐swimming (blue).

A total of 999 companions across 18 taxa were associated with hosts (Figure 3, Table 2). Most companions (73%) were free‐swimming carangids, dominated by yellowtail scad ( *Atule mate* ), which made up 38% of all observations. Other abundant carangids include unidentified scads (*Decapterus* sp.; 16%) and pilotfish (10%) (Table 2). Mean fork length of free‐swimming companions was 20 cm with measurements between 7 cm in golden trevally (*Gnathodon speciosus*) and 35 cm in Alepes scads (*Alepes* sp.) (Table 2). Suction companions formed a smaller proportion of the total companions (26%). Of these, live sharksuckers ( *Echeneis naucrates* ) and remoras ( *Remora remora* ) each accounted for 13% of observed companions. Mean fork length of suction companions was 20 cm, with measurements ranging between 2 cm (remora) and 67 cm (live sharksucker) (Table 2).

**FIGURE 4:**
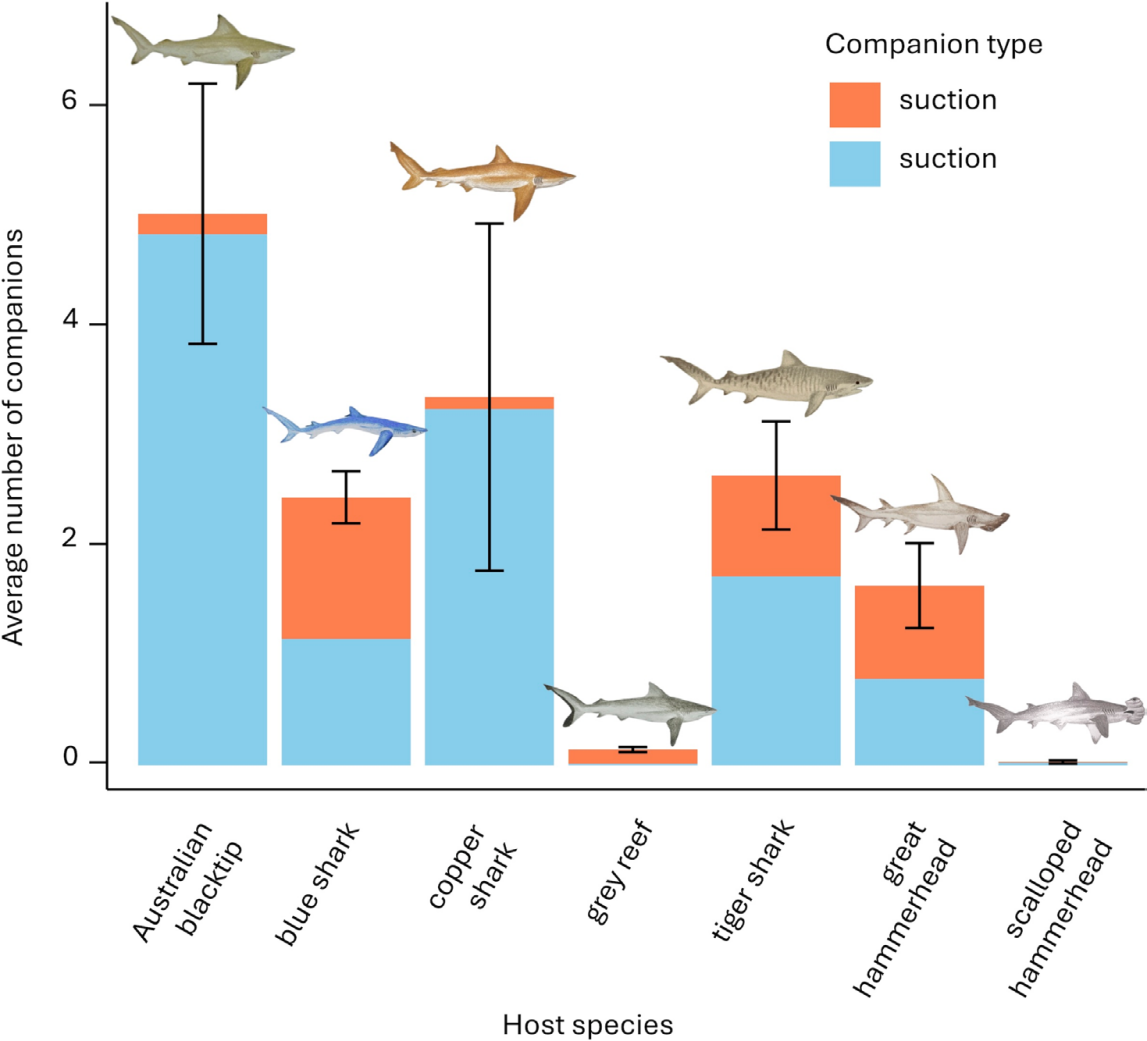
Average number of companion species per host shark species. Bars are colour‐coded by companion type with error bars representing the standard error of the mean.

Associations between companions‐hosts varied markedly across host species, revealing distinct, non‐random association patterns. The bipartite plot showed that yellowtail scad had a strong association with Australian blacktip sharks, representing 91% of their total occurrences (Figure 3). Eighty‐seven percent of unidentified scads were associated with copper sharks, while 88% of pilotfish were associated with blue sharks (Figure 3). Suction‐based companions displayed broader host ranges. Live sharksuckers associated with all host species except scalloped hammerheads and blue sharks, with near equal association between great hammerheads (26%), grey reef sharks (27%) and tiger sharks (30%) (Figure 3). Remoras associated with a lower diversity of hosts, with 76% of occurrences on blue sharks (Figure 3). Patterns of companion‐host associations also varied across companion functional groups. Copper sharks had the highest proportion of free‐swimming companions (97%), followed by Australian blacktip sharks (95%) (Figure 4). In contrast, grey reef sharks were dominated by suction‐based companions (92%), followed by great hammerheads (55%), blue sharks (53%) and tiger sharks (43%) (Figure 4).

The strength of associations was confirmed by calculating pairwise odds ratios between hosts. Odds‐ratio comparisons showed that Australian blacktip sharks were over six times more likely to host‐companions than grey reef sharks (OR = 6.72; Table S2), while copper sharks were nearly three times more likely (OR = 2.88; Table S2). In contrast, scalloped hammerheads had the lowest likelihood of hosting companions, with odds near zero relative to all other species (e.g., OR = 0.01 vs. tiger sharks; Table S2). Most hosts observed with companions carried few individuals, with 69% hosting three or fewer individuals. Only 4% of hosts with companions had more than 20 individuals. The host with the most companions was an Australian blacktip shark that carried 70 individuals. Approximately 78% of hosts observed with companions had only one companion taxon, with the maximum diversity of companions seen with a host being four taxa. Together, these patterns indicate strong heterogeneity in companion–host associations among shark species.

### 3.2 Predicting Companion Presence/Absence and Abundance

Companion presence and abundance were predicted by environmental conditions and protection status. Companion presence was best predicted, in order of contribution, by salinity (logSalinity), sea surface temperature (SST), distance to shore (logDFS), partial protection (IUCN IV/VI; DVPP) and zonal wind speed (WindU) (Table 3). Analogously, companion abundance was best predicted by salinity, primary productivity (logPP), high protection (IUCN I/II; DVHP) and zonal wind speed (Table 3). The best hurdle model was supported by a log‐likelihood of −846 and an AIC score of 1716. The model explained 22% of the variation relative to the null model. The area under the curve (AUC) was 0.84, which indicated strong discriminative ability between the presence and presence/absence of companions (Figure S1). Calibration analysis showed close agreement between predicted and observed probabilities across ten cross‐validation folds (Figure S2). MAE was interpreted relative to the mean observed abundance (3.9 companions), with an MAE of 1.5 representing a deviation of approximately 38% from the mean number of companions.

**TABLE 3:** Results of the hurdle model for companion presence and abundance. Coefficients (Estimate ± SE) and associated *p*‐values are shown for predictors retained in the zero model (occurrence probability) and the count model (abundance given presence). Significant predictors indicate the influence of environmental or management factors.

Variable	Presence			Abundance		
	Esimate	SE	*p*	Esimate	SE	*p*
windU	0.053	0.023	2.30E−02	0.098	0.029	7.27E−04
SST	0.31	0.041	1.81E−13	—	—	—
logSalinity	124	12.16	2.00E−16	111	14.55	1.99E−14
logPP	—	—	—	2.69	0.61	9.41E−06
logDFS	0.79	0.16	3.31E−07	—	—	—
DVHP	—	—	—	0.97	0.27	2.74E−04
DVPP	1.48	0.31	1.60E−06	—	—	—

**FIGURE 5:**
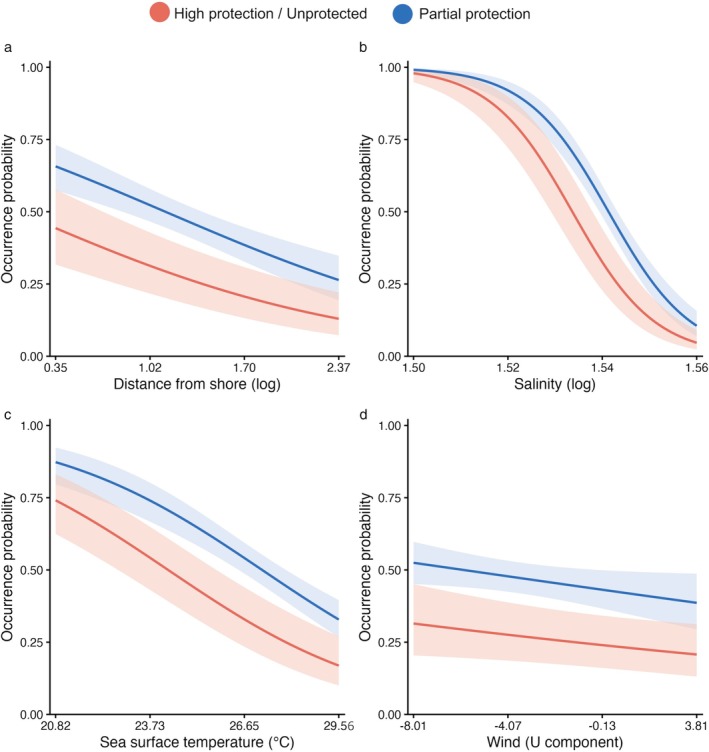
Predicted probability of companion occurrence from the zero (presence–absence) component of the hurdle model. Panels show the variation of likelihood that a shark hosts at least one companion with (a) distance from shore, (b) salinity, (c) sea surface temperature and (d) zonal wind speed (U component). Curves represent fitted model predictions with 95% confidence intervals. Blue lines correspond to deployments outside partially protected areas (no protection and high protection), and orange lines represent deployments inside partially protected areas (IUCN IV/VI). All other predictors are held at their mean values.

**FIGURE 6:**
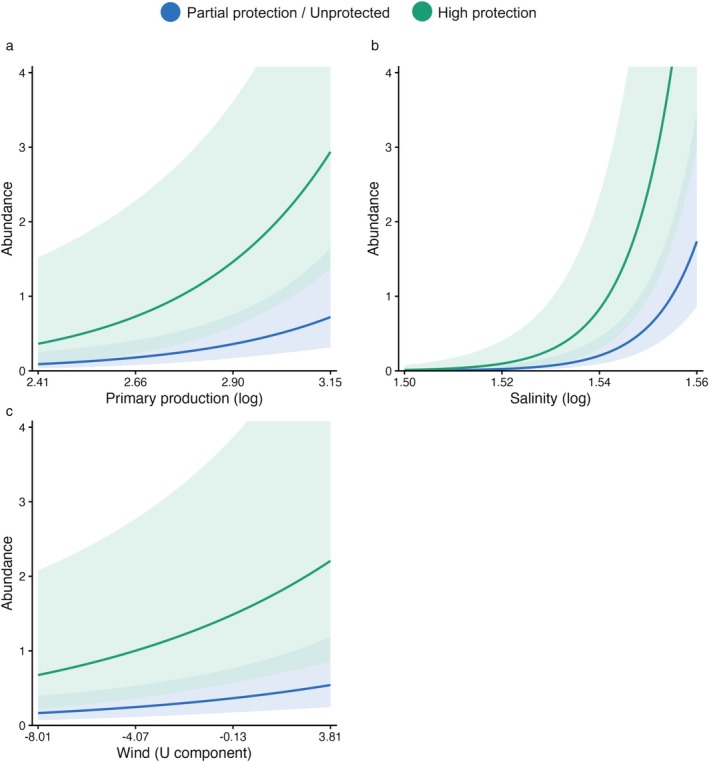
Predicted companion abundance from the count component of the hurdle model. Panels show the variation of likelihood that expected companion numbers vary with (a) primary production, (b) salinity and (c) zonal wind speed (U component). Curves represent fitted model predictions with 95% confidence intervals. Blue lines correspond to deployments outside highly protected areas (no protection and partial protection), and green lines represent deployments inside highly protected areas (IUCN I/II). All other predictors are held at their mean values.

The two components of the hurdle model differed with respect to their predictors. Zonal winds (*p* < 0.001) and higher salinity (*p* < 0.001) were significant in both models (Figures 5 and 6; Table 3). Stronger easterly winds decreased the likelihood of companion presence but increased companion abundance (*p* < 0.001 in both models) (Figures 5 and 6; Table 3). The effect of salinity was similar in that increasing salinity decreased the likelihood of companion presence but increased companion abundance (*p* < 0.001 in both models). Increasing sea surface temperature and distance to shore both decreased the likelihood of companion presence (p < 0.001 for both variables) but had no significant effect on abundance (Figure 6; Table 3). Primary productivity did not significantly affect the likelihood of companion presence; however, increasing primary productivity was associated with increased companion abundance when present (*p* < 0.001; Figure 6; Table 3). Host fork length did not significantly predict either the presence or abundance of companions in the context of other predictors (Table 3). Pearson's correlation coefficient indicated no direct linear relationship between host length and companion abundance (*r* = 0.03, *p* = 0.50).

Protection status emerged as an important predictor in both model components. Hosts in partially protected areas had a higher probability of having companions than those in unprotected or highly protected areas (*p* < 0.001; Figures 5 and 6, Table 3). Model‐based predictions showed the highest probability of companion presence in partially protected areas (74%) and the lowest in highly protected areas (45%). In unprotected areas, probability of companion presence was 54%, falling between levels observed in partial and high protection zones (Table S3). The conditional abundance of companions followed a different pattern. When present, companion abundance was higher inside highly protected areas than in unprotected or partially protected areas (*p* < 0.001; Figures 5 and 6; Table 3). Model‐based predictions showed estimates of 0.24 companions in partially protected areas, 0.26 companions in unprotected areas, and a substantially higher value of 1.06 companions in highly protected areas (Table S3). Conditional abundance did not differ between partial and unprotected areas (ratio = 0.91) but was greater in highly protected areas relative to both unprotected (ratio = 4.08) and partially protected areas (ratio = 4.49) (Table S4).

## 4 Discussion

### 4.1 Implications for Behavioural Associations and Co‐Decline

Despite the ubiquity of companion–host associations involving pelagic megafauna, our understanding of these relationships remains limited, particularly at broader spatial scales (Fuller and Parsons 2019; Gayford 2024). Most existing knowledge is derived from anecdotal observations or descriptive accounts (Fuller and Parsons 2019; Gayford 2024), leaving a limited empirical basis for evaluating how these associations vary among hosts or respond to environmental gradients. The results presented here provide the first large‐scale in situ comparative data on companion‐shark associations across species and ocean basins, allowing patterns of occurrence and abundance to be examined in relation to host identity, environmental conditions and protection status. Results demonstrate that companion‐host associations are structured, non‐random and environmentally mediated. These findings establish an ecological baseline for interpreting how environmental change, host declines, and protection status may influence the persistence of pelagic interspecies associations between sharks and their companions.

Our results raise the possibility of co‐decline among companion species, as the presence and abundance of companions varied significantly among hosts. This variation supports our first hypothesis that companions exhibit non‐random associations with hosts. Australian blacktip and copper sharks were several times more likely to host‐companions than other species, whereas scalloped hammerheads rarely had any. Such variation is ecologically important because it indicates that a few host species support a disproportionate share of companions. Patterns of companion identity further reinforce this structure. Yellowtail scad and pilotfish were observed primarily with single hosts, indicating narrow ecological specialisation, whereas live sharksuckers occurred with multiple shark species and showed more generalised associations. Comparable patterns of specialisation have been reported in other marine commensals and mutualists (Dallas and Cornelius 2015). For instance, in a study done by Dallas and Cornelius (2015), a small subset of tropical fish hosts supports the majority of parasite taxa within an ecosystem and are consequently referred to as ‘structural keystones’. Model simulations further predicted that removing those few keystone species led to disproportionate losses of parasites and triggered co‐extinction cascades among dependent species (Dallas and Cornelius 2015). Although this framework is defined in terms of parasite diversity, our results indicate a similar concentration of interaction strength, with a subset of shark hosts supporting a disproportionate abundance of companions. Together these findings suggest that sharks hosting a disproportionate share of companion individuals may function as structural keystones within pelagic communities. Consequently, the global decline in sharks (Pacoureau et al. 2021; Simpfendorfer et al. 2023) may reduce host availability and drive declines in associated companion species.

### 4.2 Environmental Sensitivity and Climate Vulnerability of Associations

Companion‐host associations were reasonably well predicted by environmental factors. Relative errors of up to 40% are considered acceptable for ecological count data with strong natural variability and zero inflation (Willmott and Matsuura 2005; Elith and Leathwick 2009), rendering our relative error of 38% within this range of acceptability. These results support the conclusion that environmental factors influence companion presence and abundance. The likelihood of observing companions decreased with distance to shore, while companion abundance rose with higher primary productivity. Nearshore waters are often characterised by higher productivity (Longhurst et al. 1995), which can support greater abundance of animals and increase opportunities for ecological interactions (Woodson and Litvin 2015), which may partially explain a higher occurrence of companion‐host interactions. Offshore environments typically support lower animal densities and fewer encounter opportunities than nearshore systems (Chamberlain et al. 2014; Palomera et al. 2007). Under such conditions, associations that confer shelter, energetic, or foraging benefits may become disproportionately valuable when they occur, potentially favouring higher persistence or detectability of companion‐host associations despite reduced encounter rates.

Companion–host associations appear sensitive to variation in ocean temperature, salinity, and wind. Ocean temperature and salinity are projected to shift substantially under climate change. Sea surface temperatures will continue to rise (Venegas et al. 2023), while tropical waters will expand into higher latitudes and become more saline (Durack and Wijffels 2010). These changes will likely alter the environmental conditions that favour companion–host associations, which are more likely to be absent in warmer, more saline environments. Previous studies show that disruption of ecological dependencies can magnify biodiversity loss through secondary co‐decline (Colwell et al. 2012; Strona and Bradshaw 2018). Modelling by Strona and Bradshaw (2018) showed that dependent associations can amplify the effects of environmental stress on planetary biodiversity by up to an order of magnitude. When viewed alongside evidence that companion–host associations respond acutely to environmental gradients, these findings imply that climate‐driven ocean change could disproportionately affect dependent species, even where hosts persist.

### 4.3 Value of Protected Areas to Companion‐Host Associations

Highly protected areas are well recognised for their capacity to increase biodiversity, abundance and fish size (Horta e Costa et al. 2025; Lester et al. 2009; Sala and Giakoumi 2017). Our results partially support that highly protected areas would support both higher frequency and greater abundances of companions than partially protected or unprotected areas. While the abundance of companions, when present, was greatest in highly protected areas, the probability of observing companions was highest in partially protected areas. Partially protected areas often show reduced ecological effectiveness compared to fully protected reserves and may perform similarly to unprotected locations in terms of abundance, likely because many human activities continue and consequently limit recovery (Costello and Ballantine 2015; Turnbull et al. 2021). Unexpectedly, the probability of companion presence in highly protected areas was equivalent to that in unprotected locations, and both were lower than in partially protected areas where companions were most frequently predicted. This result may reflect the residual nature of many highly protected areas. Resistance to MPAs from extractive sectors such as fishing and offshore oil and gas can lead to highly protected areas being established disproportionately in remote regions where fish biomass is naturally low, or in degraded regions where recovery is gradual and/or uneven (Devillers et al. 2014; O'Leary et al. 2018). It is important to note that MPA classifications used in this study reflect general levels of protection rather than species‐specific conservation objectives. Many MPAs included in this analysis were not explicitly designated to protect sharks or their associated companions, and observed effects are therefore likely to reflect indirect benefits of reduced exploitation and improved ecosystem condition rather than targeted management of these species.

As recovery of large‐bodied and long‐lived species such as sharks is typically slow (Turnbull et al. 2021), companion‐host associations may be further constrained in residual areas. In this context, prioritising strong protection of biodiversity in ecologically significant areas leads to stronger species recovery (Edgar et al. 2014; Letessier et al. 2019; Mateos and Bhatnagar 2024; Smith et al. 2025) and thus likely will assist in the re‐establishment of interspecies associations. Continued recovery of host populations makes it plausible that highly protected areas could begin to match or exceed partially protected zones in supporting the presence of companion–host associations. Given ongoing declines in sharks from overfishing and habitat degradation (Pacoureau et al. 2021; Simpfendorfer et al. 2023), our findings highlight that effective protection of host populations may be essential to prevent potential co‐decline of dependent companions in pelagic ecosystems.

Host fork length did not emerge as a significant predictor of companion‐host associations. Larger hosts have previously been theorised to support more companions due to greater surface area and resource availability (Moir et al. 2010; Gayford 2024). However, our results indicate that variation in companion associations cannot be explained by host size alone or in combination with the environmental variables included here.

### 4.4 Limitations and Directions for Future Research

This study provides the first quantitative assessment of companion–host associations across sharks, but several limitations stem from the use of BRUVS. BRUVS provide only brief and spatially constrained observations, limiting the ability to capture fine‐scale behavioural dynamics, long‐term associations, or associations that occur outside the camera's field of view. We are therefore unable to assess whether these associations extend beyond observed co‐occurrence. Future work combining BRUVS with fine‐scale tracking or animal‐borne cameras, used similarly in Fontes et al. (2023), may clarify whether companions actively track hosts or associate opportunistically. Similarly, experimental approaches such as predation‐risk assays could help disentangle which benefits pilotfish or other free‐swimming companions gain from remaining near sharks. It should also be noted that environmental effects may operate with temporal lags; however, the snapshot nature of BRUVS deployments limits our ability to explicitly model lagged responses, and environmental variables are therefore interpreted as representing prevailing conditions rather than immediate drivers. Future studies incorporating temporally explicit sampling or time‐series data would be required to directly assess lagged effects.

Our findings highlight clear host‐specific patterns, but it remains uncertain whether these associations extend beyond sharks. Large‐scale analysis across other mobile megafauna such as rays, sea turtles, or cetaceans could reveal whether similar ecological network structures in pelagic ecosystems more broadly. Examining interspecific variation among shark species not observed in this study would also improve understanding of how species and behaviour shape the formation and persistence of companion–host associations.

## 5 Conclusion

We provide rare quantitative evidence that shark‐companion associations are structured ecological associations rather than incidental co‐occurrences. Companions were not randomly distributed among hosts, with a small number of shark species supporting most associations. Such non‐random associations mirror patterns in other host–associate systems where the loss of key taxa can trigger cascading co‐declines. Environmental variables strongly influenced predictions of companion presence/absence and abundance, demonstrating that these associations are sensitive to changing ocean conditions. Most of the environmental drivers of associations are projected to shift under future climate change, putting dependent species at risk of amplified co‐decline. Differences among protection levels indicate that both partial and high protection influence these associations, but strong protection is required to sustain high companion abundances on hosts. Future work to further our understanding of interspecies associations will be crucial for protecting these relationships in a changing ocean.

## Author Contributions

**Jett K. Walker:** conceptualization (equal), data curation (lead), formal analysis (equal), investigation (lead), methodology (equal), writing – original draft (lead), writing – review and editing (equal). **Jessica J. Meeuwig:** conceptualization (equal), data curation (supporting), formal analysis (equal), funding acquisition (lead), investigation (supporting), methodology (equal), supervision (lead), writing – review and editing (equal). **Christopher D. H. Thompson:** conceptualization (equal), formal analysis (supporting), investigation (supporting), methodology (equal), supervision (supporting), writing – review and editing (equal).

## Funding

The data used in this study were collected through projects supported by the National Geographic Pristine Seas programme, the United Kingdom Foreign Commonwealth Office, the Jock Clough Marine Foundation, and the Minderoo Foundation. No specific funding was received for the present analysis.

## Conflicts of Interest

The authors declare no conflicts of interest.

## Supporting information


**Figure S1:** Model performance diagnostics for the hurdle model predicting companion abundance. Receiver operating characteristic (ROC) curve for the zero‐inflation (presence/absence) component, showing the true positive rate versus the false positive rate with 95% confidence bounds (grey shading).
**Table S1:** sample sites within the 29 global locations by year, global position (Lat, Long), total deployments (*n*), deployments with companion‐host interactions (NC), and protection status: no protection (None), partially protected (PP), and highly protected (HP).
**Table S2:** Pairwise comparisons of host shark species for the likelihood of having companion species present. Values represent odds ratios from logistic regression models, with associated 95% confidence intervals and *p*‐values. Odds ratios greater than 1 indicate a higher likelihood of companions in the first species listed compared to the second. Extremely large confidence intervals reflect high uncertainty, likely due to sparse data or quasi‐complete separation in some comparisons. Comparisons are ordered by highest to lowest odds ratios for each species comparison subsection.
**Table S3:** Model‐predicted probabilities of companion presence (presence), mean abundance given presence (mean|presence), and overall expected abundance (exp‐abund) for sharks observed in unprotected, partially protected (PP), and highly protected (HP) areas. Values are model predictions with 95% confidence intervals.
**Table S4:** Contrasts between protection levels from the hurdle model. Presence contrasts are expressed as odds ratios (ORs) for the probability of observing companions, and abundance contrasts are expressed as ratios of mean abundance given presence. Values are presented with 95% confidence intervals.

## Data Availability

Data and analysis code are available at: https://doi.org/10.5061/dryad.rr4xgxdpm.
