# Correction to “Environmental and Protection Effects of Shark‐Companion Associations Across Three Ocean Basins”

**DOI:** 10.1002/ece3.73961

**Published:** 2026-06-30

Walker, J. K. , J. J. Meeuwig , and C. D. H. Thompson . 2026. “Environmental and Protection Effects of Shark‐Companion Associations Across Three Ocean Basins.” Ecology and Evolution 16, no. 6: e73823. 10.1002/ece3.73823.42282979 PMC13249542

In the legend of Figure 4, the blue companion type category was incorrectly labeled as “suction”. It should read “free‐swimming”.

This correction relates only to the figure legend. The figure data, analyses, results, and conclusions of the article remain unchanged.

The corrected Figure 4 is provided below.

**FIGURE 4:**
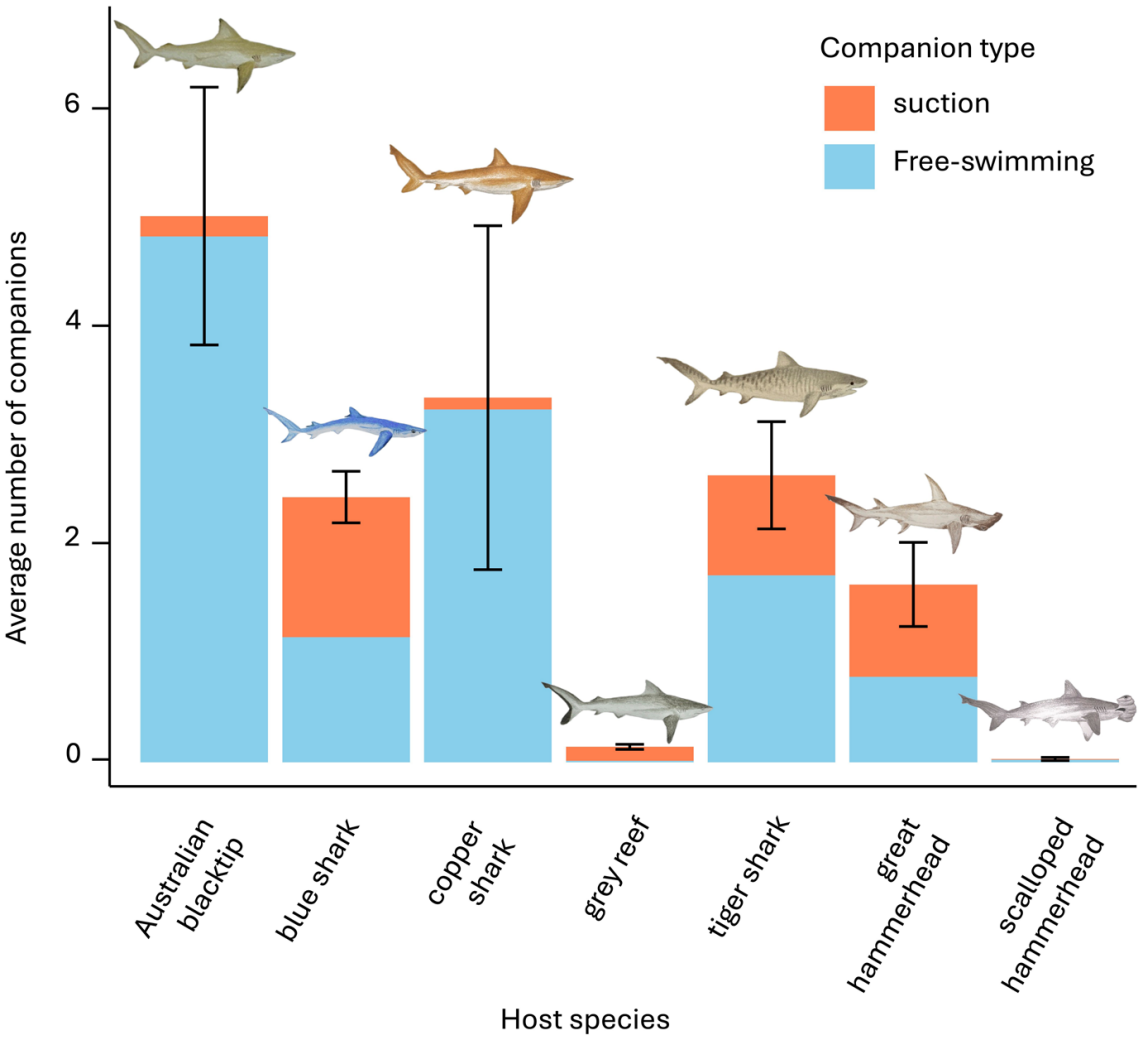
Average number of companion species per host shark species. Bars are colour‐coded by companion type with error bars representing the standard error of the mean.

We apologize for this error.

