# Supplementary material for: Environmental and Protection Effects of Shark‐Companion Associations Across Three Ocean Basins
Source: Ecol Evol. 2026 Jun 9;16(6):e73823. doi: 10.1002/ece3.73823 (PMC13249542; doi:10.1002/ece3.73823)
Supplement: Supplementary file 1 — Figure S1: Model performance diagnostics for the hurdle model predicting companion abundance. Receiver operating characteristic (ROC) curve for the zero‐inflation (presence/absence) component, showing the true positive rate versus the false positive rate with 95% confidence bounds (grey shading). Table S1: sample sites within the 29 global locations by year, global position (Lat, Long), total deployments (n), deployments with companion‐host interactions (NC), and protection status: no protection (None), partially protected (PP), and highly protected (HP). Table S2: Pairwise comparisons of host shark species for the likelihood of having companion species present. Values represent odds ratios from logistic regression models, with associated 95% confidence intervals and p‐values. Odds ratios greater than 1 indicate a higher likelihood of companions in the first species listed compared to the second. Extremely large confidence intervals reflect high uncertainty, likely due to sparse data or quasi‐complete separation in some comparisons. Comparisons are ordered by highest to lowest odds ratios for each species comparison subsection. Table S3: Model‐predicted probabilities of companion presence (presence), mean abundance given presence (mean|presence), and overall expected abundance (exp‐abund) for sharks observed in unprotected, partially protected (PP), and highly protected (HP) areas. Values are model predictions with 95% confidence intervals. Table S4: Contrasts between protection levels from the hurdle model. Presence contrasts are expressed as odds ratios (ORs) for the probability of observing companions, and abundance contrasts are expressed as ratios of mean abundance given presence. Values are presented with 95% confidence intervals. [file ECE3-16-e73823-s001.docx]

**Appendix 1: supplementary figures**

**
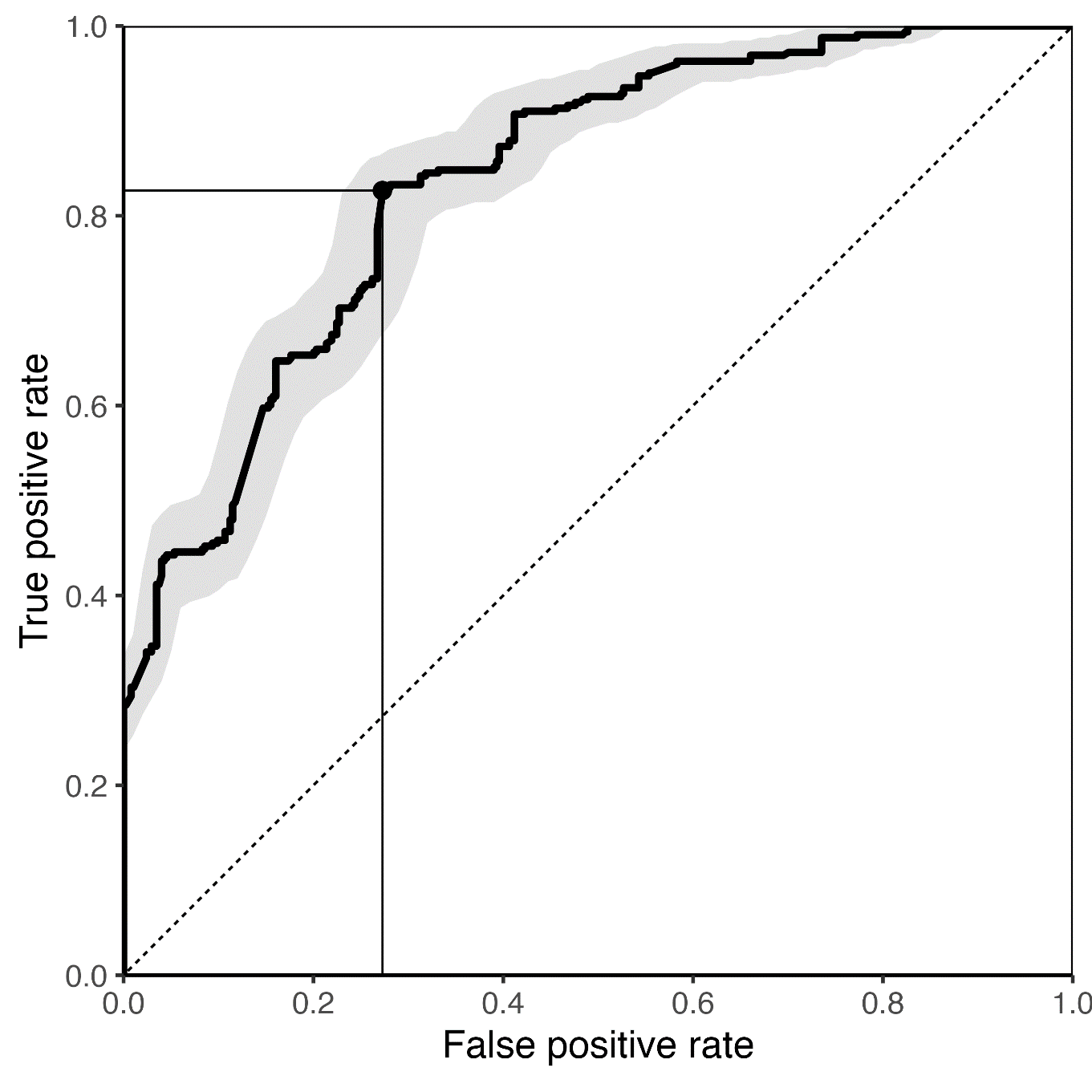
Figure S1.** Model performance diagnostics for the hurdle model predicting companion abundance. Receiver operating characteristic (ROC) curve for the zero‐inflation (presence/absence) component, showing the true positive rate versus the false positive rate with 95% confidence bounds (grey shading).





**Figure S2.** Model performance diagnostics for the hurdle model predicting companion abundance. Calibration plot of the count component, based on 10‐fold cross‐validation out-of-fold (OOF) predictions. Points show observed versus predicted values across deciles of predicted abundance; vertical bars denote binomial 95% confidence intervals, and the dashed line indicates perfect calibration.

**Appendix 2: Supplementary tables**

**Table S1.** sample sites within the 29 global locations by year, global position (Lat, Long), total deployments (n), deployments with companion host interactions (NC), and protection status: no protection (None), partially protected (PP), and highly protected (HP).

| Survey | Year | Location | Lat | Long | n | NC | Protection Status |
| --- | --- | --- | --- | --- | --- | --- | --- |
| AIG | 2017 | Ascension Island | -8.49693 | -13.98884 | 38 | 36 | None |
| AIG18 | 2018 | Ascension Island | -7.46161 | -14.22940 | 14 | 14 | None |
| AZO18 | 2018 | Azores Islands | 39.09148 | -30.18815 | 7 | 3 | None |
| BRE17 | 2017 | Bremer Bay | -34.69610 | 119.69336 | 8 | 7 | PP |
| BRE19 | 2019 | Bremer Bay | -34.71976 | 119.71851 | 10 | 6 | PP |
| CHG15 | 2015 | Chagos | -6.35665 | 72.44188 | 17 | 0 | HP |
| COCP16 | 2016 | Cocos Keeling Islands | -12.13047 | 96.82653 | 42 | 6 | None |
| COLP | 2022 | Colombia | 9.16890 | -79.72284 | 67 | 2 | None |
| FNQP17 | 2017 | Far North Queensland | -10.98539 | 143.45464 | 24 | 9 | HP |
| FNQP17N | 2017 | Far North Queensland | -11.49633 | 143.43918 | 90 | 72 | None |
| GCT18 | 2018 | Gracetown | -34.03260 | 114.77150 | 3 | 1 | PP |
| GCT19 | 2019 | Gracetown | -34.02817 | 114.77163 | 3 | 0 | PP |
| GCT20 | 2020 | Gracetown | -34.01965 | 114.78783 | 5 | 1 | HP |
| GCT22 | 2022 | Gracetown | -34.03008 | 114.78346 | 1 | 1 | PP |
| GEO17 | 2017 | Geographe Bay | -33.53675 | 115.20921 | 2 | 2 | PP |
| GEO19 | 2019 | Geographe Bay | -33.44609 | 115.25626 | 2 | 1 | HP |
| GEO20 | 2020 | Geographe Bay | -33.48191 | 115.24391 | 3 | 3 | PP |
| GEO22 | 2022 | Geographe Bay | -33.48012 | 115.24265 | 9 | 8 | HP |
| GNP19 | 2019 | Galapagos | -0.73817 | -89.44434 | 1 | 1 | PP |
| MAL18 | 2018 | Malpelo | 4.00006 | -81.61266 | 47 | 2 | PP |
| MLDP | 2023 | Maldives | -0.03418 | 73.24821 | 15 | 12 | None |
| MON18 | 2018 | Montebellos | -20.08379 | 115.35809 | 8 | 5 | PP |
| NIN16 | 2016 | Ningaloo | -21.83775 | 113.57752 | 1 | 0 | PP |
| NIN18 | 2018 | Ningaloo | -21.84015 | 113.88320 | 2 | 2 | None |
| NIN19 | 2019 | Ningaloo | -21.81724 | 113.91980 | 9 | 7 | PP |
| NIN21 | 2021 | Ningaloo | -21.81331 | 113.90122 | 3 | 1 | PP |
| NIN22 | 2022 | Ningaloo | -21.82959 | 113.89282 | 2 | 2 | None |
| NIU16 | 2016 | Niue | -19.53516 | -168.82262 | 2 | 1 | None |
| NIUP23 | 2023 | Niue | -19.35832 | -168.73257 | 20 | 12 | None |
| NWP17 | 2017 | Northwest Australian Shelf | -13.01698 | 124.40123 | 24 | 9 | PP |
| NWP18 | 2018 | Northwest Australian Shelf | -13.05369 | 124.39837 | 16 | 13 | PP |
| PAL14 | 2014 | Palau | 7.43007 | 134.45608 | 8 | 1 | None |
| PC18A | 2018 | Perth Canyon | -31.98305 | 115.12041 | 3 | 1 | PP |
| PC19A | 2019 | Perth Canyon | -31.96917 | 115.11619 | 4 | 1 | PP |
| PCA22 | 2022 | Perth Canyon | -31.96355 | 115.11185 | 2 | 1 | PP |
| RAE19P | 2019 | Recherche Archipelago | -33.81940 | 124.28698 | 14 | 0 | None |
| RAM19P | 2019 | Recherche Archipelago | -34.23301 | 123.36064 | 4 | 0 | PP |
| RAW19P | 2019 | Recherche Archipelago | -34.29173 | 122.01824 | 2 | 1 | None |
| RMIP23 | 2023 | Marshall Islands | 12.51037 | 167.81937 | 87 | 49 | None |
| RVG16 | 2016 | Revillagigedo | 19.04319 | -111.06295 | 7 | 0 | HP |
| SBP18 | 2018 | Shark Bay | -26.16718 | 113.06970 | 1 | 1 | None |
| SBP19 | 2019 | Shark Bay | -26.14747 | 113.05146 | 2 | 1 | None |
| SLIP | 2021 | Southern Line Islands | -10.37864 | -151.09471 | 32 | 12 | None |
| TR21 | 2021 | Two Rocks | -31.54291 | 115.45957 | 1 | 0 | PP |
| TRI17 | 2017 | Tristan | -37.91164 | -11.84043 | 5 | 2 | None |
| URU21 | 2021 | Uruguay | -35.68927 | -52.80337 | 2 | 2 | None |
| WANP1 | 2017 | Wandoo | -20.13940 | 116.32375 | 5 | 4 | None |
| WANP2 | 2024 | Wandoo | -20.13036 | 116.38868 | 7 | 4 | None |
| WANP3 | 2018 | Wandoo | -20.11041 | 116.46919 | 5 | 3 | None |
| WANP4 | 2018 | Wandoo | -20.09858 | 116.53441 | 5 | 1 | None |
| WANP5 | 2019 | Wandoo | -20.10546 | 116.50602 | 1 | 0 | None |
| WANP6 | 2019 | Wandoo | -20.14023 | 116.32359 | 7 | 2 | None |

**Table S2.** Pairwise comparisons of host shark species for the likelihood of having companion species present. Values represent odds ratios from logistic regression models, with associated 95% confidence intervals and p-values. Odds ratios greater than 1 indicate a higher likelihood of companions in the first species listed compared to the second. Extremely large confidence intervals reflect high uncertainty, likely due to sparse data or quasi-complete separation in some comparisons. comparisons are ordered by highest to lowest odds ratios for each species comparison subsection.

| Comparison | Odds_Ratio | Lower_95_CI | Upper_95_CI | P_value | Significance |
| --- | --- | --- | --- | --- | --- |
| *Carcharhinus brachyurus / Carcharhinus amblyrhynchos* | 2.88 | 0.39 | 21.58 | 0.0473 | * |
| *Carcharhinus tilstoni / Carcharhinus amblyrhynchos* | 6.72 | 0.14 | 320.00 | 2E-09 | *** |
| *Carcharhinus tilstoni / Carcharhinus brachyurus* | 2.33 | 0.40 | 13.66 | 0.299 | - |
| *Galeocerdo cuvier / Carcharhinus amblyrhynchos* | 30.92 | 0.00 | 6.42E+11 | 6E-14 | *** |
| *Galeocerdo cuvier / Carcharhinus brachyurus* | 10.73 | 0.00 | 1.93E+05 | 7.23E-06 | *** |
| *Galeocerdo cuvier / Carcharhinus tilstoni* | 4.60 | 0.11 | 202.00 | 5.08E-03 | ** |
| *Prionace glauca / Carcharhinus amblyrhynchos* | 41.68 | 0.00 | 1.86E+15 | 8E-14 | *** |
| *Prionace glauca / Carcharhinus brachyurus* | 14.47 | 0.00 | 6.63E+06 | 1.30E-07 | *** |
| *Prionace glauca / Carcharhinus tilstoni* | 6.20 | 0.04 | 932.00 | 1.97E-04 | *** |
| *Prionace glauca / Galeocerdo cuvier* | 1.35 | 0.37 | 4.89 | 0.996 | - |
| *Sphyrna lewini / Carcharhinus amblyrhynchos* | 0.21 | 0.17 | 0.26 | 0.0576 | - |
| *Sphyrna lewini / Carcharhinus brachyurus* | 0.07 | 0.07 | 0.08 | 2.12E-04 | *** |
| *Sphyrna lewini / Carcharhinus tilstoni* | 0.03 | 0.03 | 0.03 | 1E-08 | *** |
| *Sphyrna lewini / Galeocerdo cuvier* | 0.01 | 0.01 | 0.01 | 1E-13 | *** |
| *Sphyrna lewini / Prionace glauca* | 0.01 | 0.01 | 0.01 | 6E-14 | *** |
| *Sphyrna mokarran / Sphyrna lewini* | 46.93 | 0.00 | 9.34E+25 | 5E-09 | *** |
| *Sphyrna mokarran / Carcharhinus amblyrhynchos* | 9.86 | 0.01 | 1.51E+04 | 3E-08 | *** |
| *Sphyrna mokarran / Carcharhinus brachyurus* | 3.42 | 0.16 | 72.58 | 0.0977 | - |
| *Sphyrna mokarran / Carcharhinus tilstoni* | 1.47 | 0.45 | 4.73 | 0.966 | - |
| *Sphyrna mokarran / Galeocerdo cuvier* | 0.32 | 0.24 | 0.43 | 0.214 | - |
| *Sphyrna mokarran / Prionace glauca* | 0.24 | 0.19 | 0.30 | 0.0409 | * |

**Table S3**. Model-predicted probabilities of companion presence (presence), mean abundance given presence (mean|presence), and overall expected abundance (exp-abund) for sharks observed in unprotected, partially protected (PP), and highly protected (HP) areas. Values are model predictions with 95% confidence intervals.

| Protection level | Presence (%) | mean-abund | exp-abund |
| --- | --- | --- | --- |
| No protection | 45.9 (40.0 – 51.9) | 0.26 (0.12 – 0.56) | 0.12 (0.05 – 0.26) |
| Partially protected (PP) | 26.2 (17.9 – 37.2) | 0.24 (0.09 – 0.59) | 0.06 (0.02 – 0.17) |
| Highly protected (HP) | 55.1 (44.5 – 65.3) | 1.06 (0.37 – 2.82) | 0.59 (0.20 – 1.53) |

**Table S4**. Contrasts between protection levels from the hurdle model. Presence contrasts are expressed as odds ratios (ORs) for the probability of observing companions, and abundance contrasts are expressed as ratios of mean abundance given presence. Values are presented with 95% confidence intervals.

| Contrast (OR or ratio) | Estimate | | 95% CI |
| --- | --- | --- | --- |
| Presence: PP vs No | | 0.42 | 0.24 – 0.72 |
| Presence: HP vs No | | 1.45 | 0.87 – 3.10 |
| Presence: HP vs PP | | 3.45 | 1.81 – 8.36 |
| Mean\|presence: PP / No | | 0.91 | 0.50 – 1.65 |
| Mean\|presence: HP / No | | 4.08 | 1.90 – 8.74 |
| Mean\|presence: HP / PP | | 4.49 | 2.41 – 8.36 |
